# Veterinary World reviewer acknowledgment 2023

**DOI:** 10.14202/vetworld.2024.185-188

**Published:** 2024-01-22

**Authors:** A. V. Sherasiya, Riyaz

**Affiliations:** Veterinary World, Star, Gulshan Park, NH-8A, Chandrapur Road, Wankaner - 363621, Dist. Morbi, Gujarat, India

## Contributing reviewers

Veterinary World editorial team sincerely like to thank all of our reviewers who contributed to peer review for the journal in 2023.

Countries (57): Afghanistan - 1, Algeria - 1, Argentina - 1, Armenia - 1, Austria - 1, Bangladesh - 6, Belgium - 2, Brazil - 8, Canada - 2, Chile - 1, China - 12, Ecuador - 1, Egypt - 20, France - 3, Germany - 2, Ghana - 1, Greece - 2, Hungary - 2, India - 27, Indonesia - 3, Iran - 9, Iraq - 5, Italy - 10, Japan - 3, Jordan - 2, Kenya - 2, Kenya - 1, Lebanon - 1, Libya -1, Malaysia - 3, Mexico - 5, Morocco - 3, Nepal - 1, Nigeria - 4, Pakistan - 3, Philippines - 2, Poland - 6, Portugal - 5, Romania - 1, Russia - 1, Rwanda - 1, Saudi Arabia - 1, Slovenia - 1, South Africa - 4, Spain - 2, Sweden - 1 , Switzerland - 1, Taiwan - 3, Thailand - 7, Tunisia - 3, Turkey - 14, Uganda - 1, UK - 2, USA - 11, Venezuela - 1, Vietnam -1, West Indies - 1

**Table:** 

No.	Reviewer	Country	Reviewed articles
1.	A. Aygun	Turkey	2
2.	A. J. A. Ranjit Singh	India	1
3.	Abayli H.	Turkey	1
4.	Abdelaziz ED-DRA	Morocco	1
5.	Abdelfattah Monged selim	Egypt	1
6.	Abdul Rahman Omar	Malaysia	1
7.	Abdul Rohman	Indonesia	1
8.	Abdulatif. A. Asheg	Libya	3
9.	Agnieszka Marek	Poland	1
10.	Ahmed Abd El Tawab	Egypt	1
11.	Ahmed S Abdoon	Egypt	2
12.	Alberto Elmi	Italy	1
13.	Alejandro Hidalgo	Chile	4
14.	Alessandra Pelagalli	Italy	1
15.	Alfredo Medrano	Mexico	1
16.	Amira Taman	Egypt	1
17.	Ammar Ahmad Khan	Pakistan	1
18.	Amos Sematimba	USA	1
19.	Andrea Corda	Italy	1
20.	Anna Stanicka	Poland	1
21.	Anna Szilasi	Hungary	1
22.	Anut Chantiratikul	Thailand	4
23.	Archana Jain	India	1
24.	Arda Sozcu	Turkey	1
25.	Arda Yildirim	Turkey	1
26.	Ariadna Flores Ortega	Mexico	1
27.	Ashok Dangi	India	1
28.	Ashwani Kumar	India	1
29.	Asmaa Abdallah Darwish	Egypt	1
30.	Awanwee Petchkongkaew	Thailand	1
31.	Awatef Bejaoui	Tunisia	1
32.	Ayman Abdel-Aziz Swelum	Egypt	2
33.	Ayman Hassan Abd ElAziz	Egypt	1
34.	Azadeh Zahmatkesh	Iran	1
35.	Bahador Sarkari	Iran	1
36.	Banan Khalid Al-Baggou	Iraq	1
37.	Baratang Alison Lubisi	South Africa	3
38.	Bartosz Kieronczyk	Poland	1
39.	Beenish Zahid	Pakistan	1
40.	Belal S Obeidat	Jordan	1
41.	Belgin Siriken	Turkey	3
42.	Bettina Wollanke	Germany	1
43.	Buket Er Demirhan	Turkey	3
44.	Ceclia Maria Cruz Falcao	Brazil	1
45.	Cenk Er	Turkey	1
46.	Chao-Nan Lin	Taiwan	2
47.	Chunsheng Hou	China	1
48.	Dang Xuan Binh	Vietnam	1
49.	Daniel Pius Mdetele	South Africa	1
50.	Debasis Nayak	India	2
51.	Deepak Gola	India	1
52.	Deepan Kishore	USA	1
53.	Dhruv Nitinkumar Desai	USA	3
54.	Dina Aboelsoued	Egypt	1
55.	Dinah Seligsohn	Sweden	1
56.	Dirceu Guilherme de	Brazil	1
57.	Ebrahim Talebi	Iran	1
58.	Eduardo Jorge Boeri	Argentina	1
59.	Elena Harran	France	1
60.	Elisa Mazzotta	Italy	1
61.	ElSayed Eldeeb	Egypt	5
62.	Eman Dhahir Arif	Iraq	1
63.	Emilio A De Simone	Argentina	1
64.	Feng Shi	China	1
65.	Fernando de Freitas Fernandes	Brazil	1
66.	Florence D. Tarfa	Nigeria	2
67.	Fouad Kasim Mohammad	Iraq	5
68.	Francesco Serrapica	Italy	1
69.	Fufa Ido Gimba	Malaysia	1
70.	Gabriella Gaglio	Italy	1
71.	Genevieve Andre-Fontaine	France	1
72.	Giovanni De Benedetto	Italy	1
73.	Girija Regmi	USA	1
74.	Giulia Donato	Italy	1
75.	Giulia Sala	Italy	1
76.	Gnanavel Venkatesan	India	1
77.	Gokhlesh Kumar	Austria	1
78.	Han-Jia Lin	Taiwan	1
79.	Haroon Ahmed	Afghanistan	1
80.	Harvie P. Portugaliza	Philippines	3
81.	Hasan Meydan	Turkey	1
82.	Hasria Alang	Indonesia	4
83.	Haytham Senbill	Egypt	2
84.	Hazel Tamakan	Turkey	3
85.	Heba Ahmed Hussein Ahmed	Egypt	1
86.	Hengjie Xie	China	2
87.	HuaJi Qiu	China	3
88.	Idrees Mehraj Allaie	India	1
89.	Indrasen Chauhan	India	1
90.	Irene N. Ogali	Kenya	1
91.	Ismael Hernandez Avalos	Mexico	3
92.	J. Chen	USA	1
93.	J. Hafid	Morocco	1
94.	Jamal Gharekhani	Iran	1
95.	Janet Y. Nale	United Kingdom	1
96.	Jareerat Aiemsaard	Thailand	4
97.	Jess Mara Prez Jimnez	Spain	1
98.	Jianlin Wang	China	1
99.	João Simões	Portugal	2
100.	Joaquim Henriques	Portugal	1
101.	Jose Francisco Francisco Rivera Benitez	Mexico	1
102.	Julii Brainard	United Kingdom	2
103.	kalyan sarma	India	1
104.	Kannan Thandavan Arthanari	India	1
105.	Karim El Sabrout	Egypt	3
106.	Kaushal Kishor Rajak	India	1
107.	kekungu puro	India	1
108.	Khaled Sultan	Egypt	1
109.	Kwabena Dankwa	Ghana	1
110.	Laurent Falquet	Switzerland	1
111.	Lenin Lenin Aguirre Riofrio	Ecuador	2
112.	Lokman Galal	France	1
113.	Luiz Daniel de	Brazil	1
114.	Mabel Kamweli Aworh	Nigeria	1
115.	Mahdieh Rezaei	Iran	1
116.	Mai Mohammed Abuowarda	Egypt	1
117.	Małgorzata Ochota	Poland	1
118.	Marcelo Gottschalk	Canada	1
119.	Marco Antonio Pereira da Silva	Brazil	1
120.	Mario Gajdacs	Hungary	1
121.	Mário Manuel Dinis Ginja	Portugal	1
122.	Marko R. Cincovic	Slovenia	1
123.	Martina Gerken	Germany	1
124.	Maryam Rassouli	Iran	1
125.	Masashi Yuki	Japan	2
126.	Masato Nitta	Japan	1
127.	Mbarga Manga Joseph Arsene	Russia	1
128.	Md. Ahaduzzaman	Bangladesh	2
129.	Md. Tanvir Rahman	Bangladesh	3
130.	Megha Agarwal	USA	3
131.	Mehdi Shamsaie Mehrgan	Iran	1
132.	Michael S. Cockram	Canada	1
133.	Mihaela Niculae	Romania	5
134.	Miki Sakatani	Japan	1
135.	mireille serhan	Lebanon	1
136.	Mirosław Mariusz Michalski	Poland	1
137.	Mohamed Abdelsalam	Egypt	1
138.	Mohammad Reza Arabestani	Iran	1
139.	Mohammed A. Rohaim	Egypt	1
140.	Mohammed El-Houadfi	Morocco	1
141.	Mohammed Nooruzzaman	Bangladesh	1
142.	Mohammed S. ALhajj	Saudi Arabia	1
143.	Morteza Shams	Iran	1
144.	Mourad Ben Said	Tunisia	1
145.	Muhammad Kiggundu	Uganda	1
146.	Myriam Oudni-M’rad	Tunisia	1
147.	NadraElwgoud M. I. Abdou	Egypt	1
148.	Nele Caekebeke	Belgium	1
149.	Niaz Mahmud	USA	1
150.	Niels Vander Elst	Belgium	1
151.	Nikolina Rusenova	Bangladesh	1
152.	Noor Jamil Noor	Malaysia	1
153.	Nuh Kilic	Turkey	3
154.	Oluwasola Abayomi Adelusi	South Africa	1
155.	Panagiotis E Simitzis	Greece	5
156.	Pankaj Deka	India	1
157.	Paramanandham Krishnamoorthy	India	1
158.	Paulina Czechowicz	Poland	1
159.	Pie Ntampaka	Rwanda	1
160.	Pınar Şanlıbaba	Turkey	1
161.	Prapansak Srisapoome	Thailand	1
162.	Professor Md. Siddiqur	Bangladesh	1
163.	Qingyou Liu	China	1
164.	R. Umaya Suganthi	India	1
165.	Raquel Salazar Lugo	Venezuela	2
166.	Rashmi Rekha Kumari	India	1
167.	Reda A. Mohamed	West Indies	1
168.	Remil L. Galay	Philippines	1
169.	Rodrigo Alberto Jerez Ebensperger	Spain	4
170.	Rodrigo Almeida-Paes	Brazil	1
171.	Ronald G. Oldfield	USA	1
172.	Rute M Noiva	Portugal	1
173.	S. Silva	Portugal	1
174.	S. K. Hadjikakou	Greece	1
175.	S. K. Mondal	India	1
176.	Şafak Ulusoy	Turkey	2
177.	Salome W. KairuWanyoike	Kenya	1
178.	Sanjay Balkrishna Jadhao	India	1
179.	Seda Ozdikmenli Tepeli	Turkey	1
180.	Shahneaz Ali Khan	Bangladesh	1
181.	Shaimaa H. Negm	Egypt	1
182.	Shekhar Ramchandra Bhagwat	India	2
183.	Siva Raman	India	1
184.	Sleyman Ilek	Turkey	3
185.	Socorro Retana-Marquez	Mexico	1
186.	Somwang Lekjing	Thailand	1
187.	Song Gao	China	1
188.	Sreekumar Chirukandoth	India	1
189.	Sreenu Makkena	India	2
190.	Sucheeva Junnu	Thailand	1
191.	Sumon Ghosh	USA	1
192.	Suresh H Basagoudanavar	India	3
193.	Tahreer M. Al-Thuwaini	Iraq	1
194.	Takalani Judas Mpofu	South Africa	2
195.	Talita P. Resend	USA	1
196.	Tanko Polycarp Nwunuji	Nigeria	2
197.	ThankGod E. Onyiche	Nigeria	1
198.	Thomas Salles Dias	Brazil	3
199.	Tulsi Ram Gompo	Nepal	1
200.	V. V. S. Suryanarayana	India	1
201.	Vanessa S. Cruz	Brazil	1
202.	Varij Nayan	India	1
203.	Vincenzo Tufarrelli	Italy	1
204.	Vishal S Suthar	India	2
205.	Watchariya Purivirojkul	Thailand	1
206.	Widya Paramita Lokapirnasari	Indonesia	2
207.	Wisam S Hassan	Iraq	1
208.	Xiao-Feng Shan	China	1
209.	Xueyong Zhang	China	1
210.	Ya-Ching Lin	Taiwan	1
211.	Yahia Abbas Amin	Egypt	1
212.	Yan Yang	China	1
213.	Yasmine Hasanine Tartor	Egypt	1
214.	Ye Feng	China	1
215.	Yuexun Tian	USA	1
216.	Zahid Naseer	Pakistan	1
217.	Zahra Rouabah	Algeria	1
218.	Zaven Karalyan	Armenia	3
219.	ZhiMing Pan	China	1
220.	Zuhair Ismail	Jordan	2

